# Retraction: Structural characterization of peptides from *Locusta migratoria* manilensis (Meyen, 1835) and anti-aging effect in *Caenorhabditis elegans*

**DOI:** 10.1039/d2ra90038f

**Published:** 2022-04-28

**Authors:** Laura Fisher

**Affiliations:** Royal Society of Chemistry Thomas Graham House, Science Park, Milton Road Cambridge CB4 0WF UK advances-rsc@rsc.org

## Abstract

Retraction of ‘Structural characterization of peptides from *Locusta migratoria* manilensis (Meyen, 1835) and anti-aging effect in *Caenorhabditis elegans*’ by Hui Cao *et al.*, *RSC Adv.*, 2019, 9, 9289–9300, https://doi.org.10.1039/C9RA00089E.

The Royal Society of Chemistry hereby wholly retracts this *RSC Advances* article due to a significant amount of unattributed text overlap with articles by different author groups that were not cited, including articles published in *Phytochemistry* by Shan Su *et al.*,^1^*Journal of Functional Foods* by Elena M. Vayndorf *et al.*^2^ and *Natural Product Research* by Hui Ai *et al.*^3^

Jie Liu agrees to the retraction. The other authors have been informed but have not responded to any correspondence regarding the retraction.

Signed: Laura Fisher, Executive Editor, *RSC Advances*

Date: 29th March 2022

## Supplementary Material
